# Analysis of the predictive potential of good clinical response of plasma levels of clozapine in patients with resistant schizophrenia in an area of southern Spain

**DOI:** 10.1192/j.eurpsy.2023.959

**Published:** 2023-07-19

**Authors:** L. I. Muñoz-Manchado, F. González-Saiz, J. I. Pérez-Revuelta, N. Laherrán-Cantera, R. J. Pardo-Velasco

**Affiliations:** 1 Mental Health Unit, UGC North of Cadiz, Mental Health Inpatient Unit, General Hospital, Jerez de la Frontera. Fundación Biomédica de Cádiz.; 2 Mental Health Unit, Student of the university master’s degree in initiation to mental health research, University of Cadiz, Jerez de la Frontera, Cádiz., Spain

## Abstract

**Introduction:**

Resistant schizophrenia is a schizophrenia subtype characterized by a non-ability to respond to an appropriate antipsychotic treatment in dosage and duration by the patients. These patients show a lower prognostic and symptomatology. The unique drug which has shown efficacy for resistant schizophrenia treatment is clozapine, which is effective in suicide and aggressive behaviour prevention too. Whereas clozapine has numerous and serious adverse effects such as agranulocytosis risk. Because of this, and for guaranteeing an accurate diagnosis of resistant schizophrenia, distinguishing this from pseudo-resistance due to a poor tracing of schizophrenia, clozapine’s plasmatic levels monitoring is recommended in Spain by many clinical practise-guidelines.

**Objectives:**

This studio has the objective of determining if altered clozapine’s plasmatic levels have predictive potential of therapeutical response and answering what clinical and sociodemographic variables are associated to these anormal plasmatic levels.

**Methods:**

In this work, a cross-sectional observational study was carried out in which clinical and sociodemographic data obtained by the Mental Health Unit of the Jerez de la Frontera University Hospital were collected within the research project entitled: "Role of social cognition as a factor psychosocial functioning of the schizophrenic patient” (ECOFUN), of all the participating patients (in total the sample was 141 patients, of which 40 are in treatment with clozapine).

**Results:**

The sample of patients has a mean age of 44 years and medium-high educational levels. The vast majority are men and do not currently consume substances of abuse, and when this consumption occurs, tobacco and alcohol are the most consumed substances. Their total scores on the PANSS and Markova Barrios scales are generally very disparate, but with average values of 55 and 16. It has been obtained as results that there is no significant statistical correlation between the plasma levels of clozapine and the values of the PANSS scale and its subscales in the patients. On the other hand, patients treated with clozapine would present clinical and sociodemographic characteristics practically identical to those of patients treated with other antipsychotics, especially their values on the PANSS scale. In addition, plasma levels of clozapine are correlated, although not significantly, with an improvement in the positive symptomatology of schizophrenia.

**Conclusions:**

As a conclusion, unusually higher values of clozapine are correlated significantly with lower values in positive symptomatology in schizophrenia, but plasmatic levels are not correlated significantly with values of PANSS scale.

**Disclosure of Interest:**

None Declared

